# To Assess the Association between Glucose Metabolism and Ectopic Lipid Content in Different Clinical Classifications of PCOS

**DOI:** 10.1371/journal.pone.0160571

**Published:** 2016-08-09

**Authors:** Christian S. Göbl, Johannes Ott, Latife Bozkurt, Michael Feichtinger, Victoria Rehmann, Anna Cserjan, Maike Heinisch, Helmut Steinbrecher, Ivica JustKukurova, Radka Tuskova, Michael Leutner, Elisabeth Vytiska-Binstorfer, Christine Kurz, Andrea Weghofer, Andrea Tura, Christian Egarter, Alexandra Kautzky-Willer

**Affiliations:** 1 Department of Obstetrics and Gynecology, Division of Gynecologic Endocrinology and Reproductive Medicine, Medical University of Vienna, Vienna, Austria; 2 Department of Internal Medicine III, Division of Endocrinology and Metabolism, Unit of Gender Medicine, Medical University of Vienna, Vienna, Austria; 3 High Field Magnetic Resonance Centre of Excellence, Medical University of Vienna, Vienna, Austria; 4 Metabolic Unit, Institute of Neuroscience, National Research Council, Padova, Italy; John Hopkins University School of Medicine, UNITED STATES

## Abstract

**Aims:**

There are emerging data indicating an association between PCOS (polycystic ovary syndrome) and metabolic derangements with potential impact on its clinical presentation. This study aims to evaluate the pathophysiological processes beyond PCOS with particular focus on carbohydrate metabolism, ectopic lipids and their possible interaction. Differences between the two established classifications of the disease should be additionally evaluated.

**Methods:**

A metabolic characterization was performed in 53 untreated PCOS patients as well as 20 controls including an extended oral glucose tolerance test (OGTT, to assess insulin sensitivity, secretion and ß-cell function) in addition to a detailed examination of ectopic lipid content in muscle and liver by nuclear magnetic resonance spectroscopy.

**Results:**

Women with PCOS classified by the original NIH 1990 definition showed a more adverse metabolic risk profile compared to women characterized by the additional Rotterdam 2003 phenotypes. Subtle metabolic derangements were observed in both subgroups, including altered shapes of OGTT curves, impaired insulin action and hyperinsulinemia due to increased secretion and attenuated hepatic extraction. No differences were observed for ectopic lipids between the groups. However, particularly hepatocellular lipid content was significantly related to clinical parameters of PCOS like whole body insulin sensitivity, dyslipidemia and free androgen index.

**Conclusions:**

Subtle alterations in carbohydrate metabolism are present in both PCOS classifications, but more profound in subjects meeting the NIH 1990 criteria. Females with PCOS and controls did not differ in ectopic lipids, however, liver fat was tightly related to hyperandrogenism and an adverse metabolic risk profile.

## Introduction

Polycystic Ovary Syndrome (PCOS) represents a common endocrine disorder affecting about 9 to 18 percent of women in their reproductive lifespan [1]. While in the last decades investigations have highlighted different underlying mechanisms involved in the pathogenesis of the disease its clinical definition is still controversially discussed [2, 3]. There are mainly two classifications used in clinical practice, the 1990 published National Institute of Child Health and Human Disease of the United States National Institutes of Health (NIH) Meeting criteria and the 2003 revised Rotterdam criteria. The latter recommend diagnosis of PCOS if two out of three cardinal features are available: oligo- or anovulatiuon, clinical or biochemical hyperandrogenism and polycystic ovaries [4]. In contrast the NIH criteria are more restrictive, defining PCOS by chronic anovulation and hyperandrogenism (regardless of polycystic ovarian morphology) [5], with consequently lower prevalence but more severe clinical presentation [1, 6].

In addition to reproductive features of the syndrome, previous studies have demonstrated an association between PCOS and derangements in glucose metabolism, particularly impaired insulin action and compensatory hyperinsulinemia [6, 7, 8]. These alterations may underlie the specific hormonal and reproductive changes observed in some phenotypes of the syndrome [9] and thus potentially affect its clinical presentation. The pathophysiological mechanisms beyond insulin resistance are not fully explained, however, triglyceride accumulation in nonadipose tissue (e.g. skeletal muscle) might play a pivotal role [10, 11, 12]. Muscular insulin resistance accompanied by hyperinsulinemia may further promote hepatic de novo lipogenesis in young and otherwise healthy people, resulting in dyslipidemia and nonalcoholic fatty liver disease (NAFLD) [12], which is a very frequently observed condition in females with PCOS [13, 14, 15]. Actually, there is only limited data on ectopic lipid content in females with PCOS available, with some evidence indicating elevated intrahepatocellular lipids assessed by nuclear magnetic resonance (NMR) spectroscopy, specifically in phenotypes characterized by hyperandrogenism [16].

Therefore, the purpose of this study is to assess early pathophysiological characteristics of carbohydrate metabolism (i.e. glucose, insulin, and C-peptide dynamics during an oral glucose tolerance test (OGTT), insulin sensitivity, secretion, extraction and ß-cell function) and their association with hepatocellular and intramyocellular lipid content with particular focus on the clinical classification of PCOS. It is hypothesized that PCOS phenotypes primarily defined by the NIH criteria are associated with more adverse alterations and glucometabolic risk factors than those phenotypes additionally suggested by the more recent Rotterdam criteria or healthy controls.

## Materials and Methods

### Study participants

In this study caucasian females with newly diagnosed and untreated PCOS (n = 53) were consecutively recruited among women visiting our endocrinology outpatient department (Department of Obstetrics and Gynecology, Division of Gynecologic Endocrinology and Reproductive Medicine, Medical University of Vienna) between September 2012 and July 2015. PCOS was diagnosed if two out of three criteria were present: Ovulatory dysfunction (<8 hemoragic episodes per year), clinical or biochemical hyperandrogenism (Ferriman-Gallway score (FGS) >7 or total testosterone >0.48 ng/ml), as well as polycystic ovary morphology in ultrasound (≥12 follicles). Four different phenotypes of PCOS were summarized according to the NIH (PCOS-NIH; phenotype A: ovulatory dysfunction + hyperandogenism + polycystic ovaries or phenotype B: ovulatory dysfunction + hyperandrogenism) as well as the Rotterdam criteria, representing two classical and two newer phenotypes (PCOS-ROT; phenotype C: ovulatory dysfunction + polycystic ovary or phenotype D: hyperandrogenism + polycystic ovary). Women with infectious, autoimmune or malignant disorders, lipid modulating drugs, overt type 2 diabetes, metformin or other antidiabetic drugs or with diseases affecting reproductive function (with exception of PCOS) were excluded from this study. One subject (PCOS-ROT group) with newly diagnosed type 2 diabetes was also excluded. None of these females received any pharmacotherapeutic treatment for PCOS before or during the examinations. In addition, a total of n = 20 women (free of any acute or chronic diseases) recruited were included as control group. 10 controls used systemic hormonal contraceptive agents during the study period.

The study was approved by the Ethics Committee of the Medical University of Vienna and performed in accordance with the Declaration of Helsinki. All participants gave written informed consent to participate in this study.

### Laboratory and experimental methods

To obtain a detailed metabolic classification of the study population several experimental assessments were performed including serum lipids (fasting state) as well as an extended 2h-75g OGTT with measurements of glucose, insulin and C-peptid at fasting state, as well as 30’, 60’, 90’ and 120’ after ingestion. Androgen profile was routinely assessed at cycle start or after progesterone application. In a subgroup of n = 17 PCOS women with ovulatory dysfunction androgen profiling was performed at any time. However, this subgroup did not differ in terms of androgens (total testosterone: 0.50 ng/ml [0.41–0.65] vs. 0.42 ng/ml [0.30–0.61], p = 0.18; androstendione: 3.4 ng/ml [2.9–4.5] vs. 3.3 ng/ml [2.0–4.7], p = 0.254; DHEAS: 2.6 μg/ml [2.2–3.4] vs. 2.3 μg/ml [1.8–4.0], p = 0.724) or SHBG (35 nmol/l [26–57] vs. 44 nmol/l [28–64], p = 0.601), corresponding to the marginal variation of testosterone during the menstrual cycle. All laboratory parameters were measured according to the international standard laboratory methods at our certified Department of Medical and Chemical Laboratory Diagnostics (http://www.kimcl.at). The free androgen index (FAI) was calculated as the ratio of total testosterone (mmol/l) × 100 and SHBG according to [17]. Moreover, parameters of body composition (body mass index (BMI) and waist circumference (WC)) were additionally assessed.

Ectopic lipid content including hepatocellular and intramyocellular lipids (Soleus and Tibialis anterior muscle, right leg) were measured in supine position by ^1^H NMR spectroscopy based on previously described methods [18] on a 3.0 Tesla Magnetom Trio Siemens System at fasting state. Hepatocellular lipids were measured by STEAM sequence (TR = 2 s, TE = 10 ms, 4 averages, no water suppression) during single breath hold by placing the volume of interest (3cm×3cm×2cm) within the right lateral liver lobe. Hepatic lipid content was calculated from the sum intensities of methylene- (CH_2_; 1.3 ppm) and methyl- (CH_3_, 0.9 ppm) resonance lines and expressed as percent of total ^1^H MRS signal (water + lipids). T_1_ and T_2_ relaxation correction was performed using the T_1_ and T_2_ values measured at 3T. Intramyocellular lipids were measured by STEAM sequence (TR = 2 s, TE = 20 ms) with 16 averages in soleus muscle and 32 averages in tibialis anterior muscle within 1.2×1.2×2 cm^3^ voxel. Intramyocellular lipid content was calculated from the ratio of area of methylene groups signal of intramyocellular lipids (1.2–1.3 ppm) to that of water following relaxation correction as percent of tissue water MRS signal.

### Calculations

Total body insulin sensitivity during the OGTT was assessed by the composite index (ISI-Comp) [19], which is a very frequently used and validated OGTT based method in females with PCOS, in addition to the homeostasis model assessment of insulin resistance (HOMA-IR) [20], representing an approximate of hepatic insulin resistance. Insulin secretion was assessed by using the insulinogenic index to describe early (first phase) insulin response to glucose challenge (Δinsulin 0–30 min/Δglucose 0–30 min) [21]. In addition, we used a modified insulinogenic index, calculated as the quotient of the areas under the concentration curves of insulin and glucose during the OGTT (AUC-insulin/AUC-glucose 0–120’, μU/mg) to assess total posthepatic insulin secretion, as well as AUC-insulin/AUC-glucose 60–120’ (μU/mg) to estimate late phase insulin secretion [22]. The respective AUCs of glucose, insulin and C-peptide during the OGTT were calculated by using the trapezoidal rule. The amount of fasting and total hepatic insulin extraction during the OGTT was assessed as 1-(fasting insulin/fasting C-peptide) and 1-(AUC-insulin/AUC-C-peptide), respectively [23].

The association between total posthepatic insulin secretion (AUC-Glucose/AUC-Insulin, 0–120’) and insulin sensitivity (ISI-Comp) was assessed by using fractional polynomials (using a backward selection algorithm to find a suitable power transformation according to [24]). The hence derived power transformation of ISI-Comp [(x/10)^-1^] was suitable to describe the association with insulin secretion in all subgroups (with a R-squared of 0.52, 0.75 and 0.57 for controls, PCOS-NIH and PCOS-ROT, respectively). The oral disposition index (a measurement of ß-cell function) was subsequently calculated as the difference between observed and estimated values (i.e. the residuals from the estimated regression function) after excluding a possible interaction between PCOS subgroups.

The fatty liver index, a validated algorithm based on BMI, WC, TG and gamma-glutamyl transferase, was calculated according to [25].

### Statistical analysis

Categorical variables were summarized by counts and percentages. Continuous scaled variables were summarized by medians and interquartile ranges (IQR). Due to skewed distribution of some parameters (and particularly of NMR parameters) we used rank based procedures for group based comparisons (i.e. nonparametric comparisons for relative effects, which have much less assumption on the underlying distribution function as compared to the classical parametric approaches [26]). Thereby, two groups (e.g. PCOS vs. controls) were compared by using the method proposed by Brunner and Munzel [27]. For k = 3 groups (controls vs. PCOS-NIH vs. PCOS-ROT) two sample comparisons were performed on global ranks if the global null hypothesis was rejected (comparable to Fisher protected LSD in the classical ANOVA). An adjustment for demographic variables (such as age BMI and WC) was performed by using the proportional odds model. PCOS phenotypes (4 phenotypes) were compared with the control group by using Dunnett’s procedure to achieve a 95% coverage probability. Bivariate correlations between ordinal and metric scaled variables were assessed by Spearman’s rank correlation (rho). Glucose, insulin and C-peptide dynamics during the OGTT were visualized by plotting individual data (spaghetti plot). As single measurements of the OGTT examination were missing for some observations (occurred in n = 5 subjects) we performed multivariate imputations by chained equations (including the information of all available OGTT data in addition to PCO status) and estimated the missing values by the average of m = 50 complete data sets. Details of missing OGTT data are provided in Table 1.

**Table 1:** Summary of missing OGTT data in PCOS and controls.

PCOS	Glucose	Insulin	C-Peptide	Controls	Glucose	Insulin	C-Peptide
	OGTT	OGTT	OGTT		OGTT	OGTT	OGTT
*Time of OGTT samplimg*	*Missing*	*Missing*	*Missing*	*Time of OGTT samplimg*	*Missing*	*Missing*	*Missing*
**0'**	0	0	0	**0'**	0	0	0
**30'**	1	1	1	**30'**	0	0	0
**60'**	0	1	1	**60'**	0	0	0
**90'**	3	3	3	**90'**	1	1	1
**120'**	0	0	0	**120'**	0	0	0
**Total number of missing data**	4 of 265	5 of 265	5 of 265	**Total number of missing data**	1of 100	1 of 100	1 of 100

Statistical analysis was performed with R (V3.2.2) and contributed packages (particularly the R-packages "mice" for multiple imputations, “mfp”, "nparcomp" and “rms” for data analysis as well as "lattice", "beeswarm" and “corrplot” for visualizations) [28]. The two-sided significance level was set to 0.05. However, p-values were interpreted in an explorative manner and there was no further adjustment for multiplicity as not otherwise indicated.

## Results

### Descriptive characteristics of females with PCOS

Out of 53 females with PCOS included in this study, 46 (86.8%) showed ovulatory dysfunction, 42 (79.2%) clinical or biochemical hyperandrogenism and 46 (86.8%) polycystic ovary morphology in ultrasound. Accordingly, 35 (66.0%) females met the NIH (PCOS-NIH), and 18 (34.0%) the additional Rotterdam criteria (PCOS-ROT). Clinical characteristics of the study sample are provided in Table 2. As compared to healthy controls, females with PCOS (particularly in the PCOS-NIH subgroup) showed significantly higher BMI and WC, dyslipidemia and a characteristic sex hormone profile with clinical or biochemical hyperandrogenism as well as decreased SHBG (sex hormone binding globulin) levels. The “metabolic syndrome” (according to the NCEP-ATP III criteria) was present in five subjects (PCOS-NIH: 3; PCOS-ROT: 2) and two subjects showed impaired glucose tolerance (i.e. 2h post load glucose levels ≥140 mg/dl).

**Table 2:** Basic characteristics of the study sample.

	PCOS-Total	PCOS-NIH	PCOS-ROT	Controls	p-value§

Age (years)	25 (22–31)	25 (22–29)	26 (22–33)	23 (23–25)	0.370
BMI (kg/m2)	25 (22–30)*	25 (23–30)*	23 (21–30)	21 (20–24)	0.001
Waist (cm)	84 (75–94)	89 (80–95)*†	76 (70–84)	78 (75–85)	0.014
TG (mg/dl)	79 (63–95)	75 (62–92)	85 (64–96)	99 (78–110)	0.094
TC (mg/dl)	179 (153–198)	179 (153–199)	178 (163–198)	170 (158–180)	0.602
LDL-C (mg/dl)	102 (77–124)*	99 (78–126)	103 (78–120)	81 (70–87)	0.135
HDL-C (mg/dl)	57 (44–67)*	55 (44–65)*	59 (46–72)	68 (60–78)	0.011
LH (mU/ml)	8.1 (5.9–12.1)*	8.5 (6.5–14.7)*	7.0 (4.9–9.0)	4.0 (1.6–6.8)	<0.001
FSH (mU/ml)	5.5 (4.9–6.5)	5.6 (5.0–6.7)	5.3 (4.8–6.2)	5.0 (2.5–6.5)	0.401
SHBG (nmol/l)	35 (27–58)*	34 (23–53)*	57 (33–81)*	94 (53–136)	<0.001
FG-Score	10.0 (5.0–15.0)*	12.0 (8.5–15.5)*†	4.0 (3.0–9.8)*	0.0 (0.0–0.5)	<0.001
Tot-Test (ng/ml)	0.44 (0.32–0.63)*	0.51 (0.41–0.67)*†	0.33 (0.22–0.41)	0.37 (0.27–0.46)	0.003
FAI	4.2 (2.0–6.5)*	5.3 (3.5–6.7)*†	2.0 (1.3–3.0)	1.6 (0.5–2.9)	<0.001
ANST (ng/ml)	3.4 (2.2–4.7)*	3.9 (2.7–5.0)*†	2.6 (2.0–3.4)	2.1 (1.7–2.9)	0.006
DHEAS (μg/ml)	2.5 (1.9–4.0)	2.9 (2.2–4.0)	2.1 (1.7–3.2)	2.1 (1.5–3.4)	0.178

Data are median and interquartile range (IQR) for controls, patients with PCOS (total group) as well as for subgroups classified by NIH 1999 criteria (PCOS-NIH) and patients additionally identified by the Rotterdam 2003 criteria (PCOS-ROT). BMI, body mass index; WC, waist circumference; TG, triglycerides; TC, total cholesterol; LDL-C, low-density lipoprotein cholesterol; HDL-C, high-density lipoprotein cholesterol; LH, luteinizing hormone, FSH, follicle stimulating hormone; SHBG, sex hormone binding globulin; FG-Score, Ferriman-Gallway score; Tot-Test, total testosterone; FAI, free androgen index; ANST, androstendione; DHEAS, dehydroepiandrostendione.

* vs. controls: p<0.05

† PCOS-NIH vs. PCOS-ROT: p<0.05

§ test for global hypothesis: controls vs. PCOS-NIH vs. PCOS-ROT: p<0.05.

### Assessment of OGTT dynamics and insulin resistance

**Fig 1:**
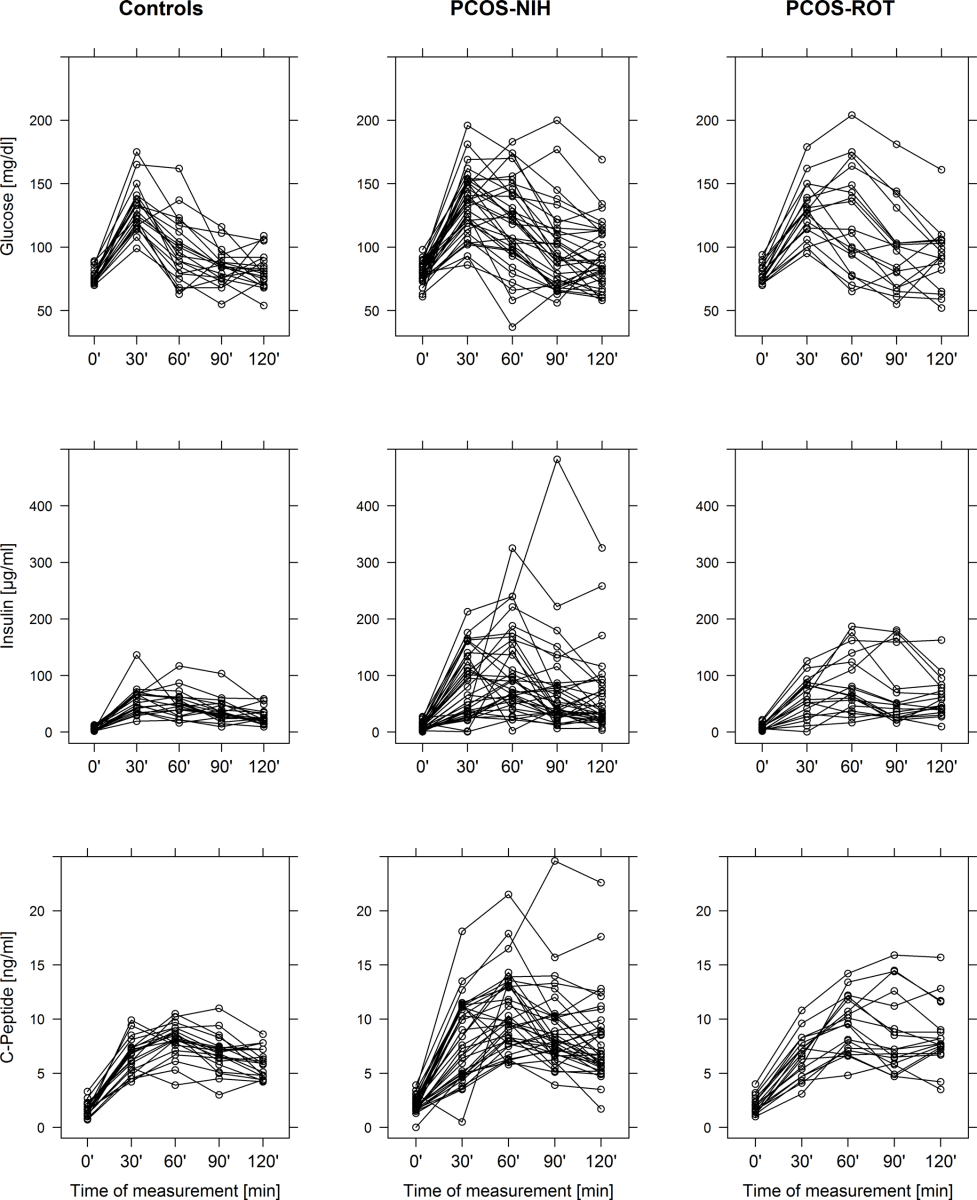
Spaghetti plots of plasma glucose, insulin and C-Peptide dynamics during a 2h-oral glucose tolerance test (OGTT) in control subjects, patients with PCOS classified by NIH 1999 criteria (PCOS-NIH) as well as patients with PCOS additionally classified by the Rotterdam 2003 criteria (PCOS-ROT).

Fig 1 revealed group specific differences in glucose, insulin and C-peptide dynamics during the OGTT. Both PCOS classifications showed a significant delay in reaching the maximum concentrations of glucose and insulin as compared to healthy controls. While postprandial glucose levels (maximum concentrations as well as AUCs) were comparable between the groups, the maximum concentrations of insulin were significantly increased in both PCOS-NIH and PCOS-ROT as compared to healthy women. Moreover, females with PCOS-NIH were further characterized by increased AUC of insulin and C-Peptide (details are provided in Table 3).

**Table 3:** Comparision of glucometabolic parameters.

	PCOS-Total	PCOS-NIH	PCOS-ROT	Controls	p-value§

Fasting-G (mg/dl)	80 (74–86)	80 (75–86)	77 (74–84)	76 (73–79)	0.118
Max-G (mg/dl)	137 (115–153)	138 (119–154)	133 (114–150)	131 (119–139)	0.624
time-max-G (min)	30 (30–60)*	30 (30–60)*	30 (30–60)*	30 (30–30)	0.016
AUC-G (g/dl)	13 (11–15)	13 (11–15)	13 (11–15)	12 (11–12)	0.157
Fastig-I (μU/ml)	7.8 (5.6–12.4)*	7.8 (5.7–12.2)*	8.5 (5.0–12.4)*	4.4 (2.7–8.9)	0.016
Max-I (μU/ml)	93 (61–162)*	108 (66–161)*	82 (55–153)*	56 (44–68)	<0.001
time-max-I (min)	60 (30–90)*	60 (30–90)*	60 (60–90)*	45 (30–60)	0.018
AUC-I (mU/ml)	6.7 (4.4–11.2)*	7.3 (4.7–10.9)*	5.9 (3.7–11.3)	4.4 (3.6–5.6)	0.004
Fastig-CP (ng/ml)	2.0 (1.6–2.5)*	2.0 (1.7–2.6)*	2.0 (1.4–2.4)	1.6 (1.2–2.0)	0.016
Max-CP (ng/ml)	10.5 (8.2–12.9)*	11.1 (8.6–13.2)*	9.6 (7.7–12.5)	8.6 (7.8–9.4)	<0.001
time-max-CP (min)	60 (60–90)*	60 (60–90)†	90 (60–120)*	60 (60–60)	0.002
AUC-CP (ng/ml)	904 (716–1110)*	921 (741–1129)*	845 (676–1083)	777 (708–841)	0.018
HOMA-IR	1.6 (1.0–2.4)*	1.6 (1.0–2.3)*	1.7 (0.9–2.5)*	0.9 (0.5–1.7)	0.015
ISI-Comp	5.9 (3.3–9.1)*	5.6 (3.6–8.5)*	6.3 (3.3–10.9)*	10.3 (6.7–14.0)	0.001
Sec-Early (μU/mg)	113 (64–200)	113 (60–215)	113 (70–145)	93 (67–127)	0.475
Sec-Late (μU/mg)	62 (42–88)*	63 (43–88)*	58 (41–86)*	42 (34–56)	0.009
Sec-Total (μU/mg)	56 (32–84)*	64 (33–89)*	55 (33–78)	41 (28–53)	0.012
FHIE (%)	0.91 (0.90–0.93)*	0.91 (0.90–0.93)*	0.91 (0.90–0.92)*	0.94 (0.92–0.95)	0.008
THIE (%)	0.84 (0.79–0.87)*	0.84 (0.79–0.86)*	0.85 (0.78–0.88)*	0.88 (0.86–0.89)	0.004

Data are median and interquartile range (IQR) for controls, patients with PCOS (total group) as well as for subgroups classified by NIH 1999 criteria (PCOS-NIH) and patients additionally identified by the Rotterdam 2003 criteria (PCOS-ROT). Fasting-G, Fasting-I Fasting-CP, fasting glucose, insulin, C-peptide; Max-G, Max-I, Mac-CP, maximum concentration of glucose, insulin, C-peptide; time-max-G, time-max-I, time-max-CP, time to reach the maximum concentrations of glucose, insulin, C-peptide; AUC-G, AUC-I, AUC-CP, area under the concentration curve (120’) of glucose, insulin and C-peptide; HOMA-IR, homeostatic model assessment of insulin resistance; ISI-Comp, Composite index; early (sec-early: Δinsulin 0–30 min/Δglucose 0–30 min), late (sec-late: AUC-Insulin/AUC-Glucose [60–120 min]) and total insulin secretion (sec-total: AUC-Insulin/AUC-Glucose [0–120 min]); FHIE, hepatic insulin extraction at fasting; THIE, total hepatic insulin extraction during the OGTT.

* vs. controls: p<0.05

† PCOS-NIH vs. PCOS-ROT: p<0.05

§ test for global hypothesis: controls vs. PCOS-NIH vs. PCOS-ROT: p<0.05.

Impaired insulin sensitivity was prevalent in both PCOS subgroups in terms of decreased ISI-Comp, which was significantly related to dyslipidemia (triglycerides: rho = -0.35, p = 0.011; LDL-cholesterol: rho = -0.35, p = 0.010; HDL-cholesterol: rho = 0.55, p<0.001) and obesity (BMI: rho = -0.59, p<0.001, WC: rho = -0.50, p<0.001). Whole body insulin sensitivity was additionally associated with SHBG (rho = 0.62, p<0.001) and consequently inversely related to FAI (rho = -0.55, p<0.001). This association was also confirmed in a sensitivity analysis after excluding n = 10 females with systemic contraceptive agents.

A higher amount of insulin resistance was also prevalent in n = 11 PCOS-ROT females with normoandrogenemia (ISI-Comp: 5.9, IQR 3.0–10.7) as compared to healthy controls (10.3, IQR 6.7–14.0, p = 0.017). Moreover, the association of insulin resistance and PCOS was shown to be independent of age and BMI (p = 0.023) or WC (p = 0.003) after covariate adjustment.

### Assessment of ß-cell function and hepatic insulin extraction

**Fig 2:**
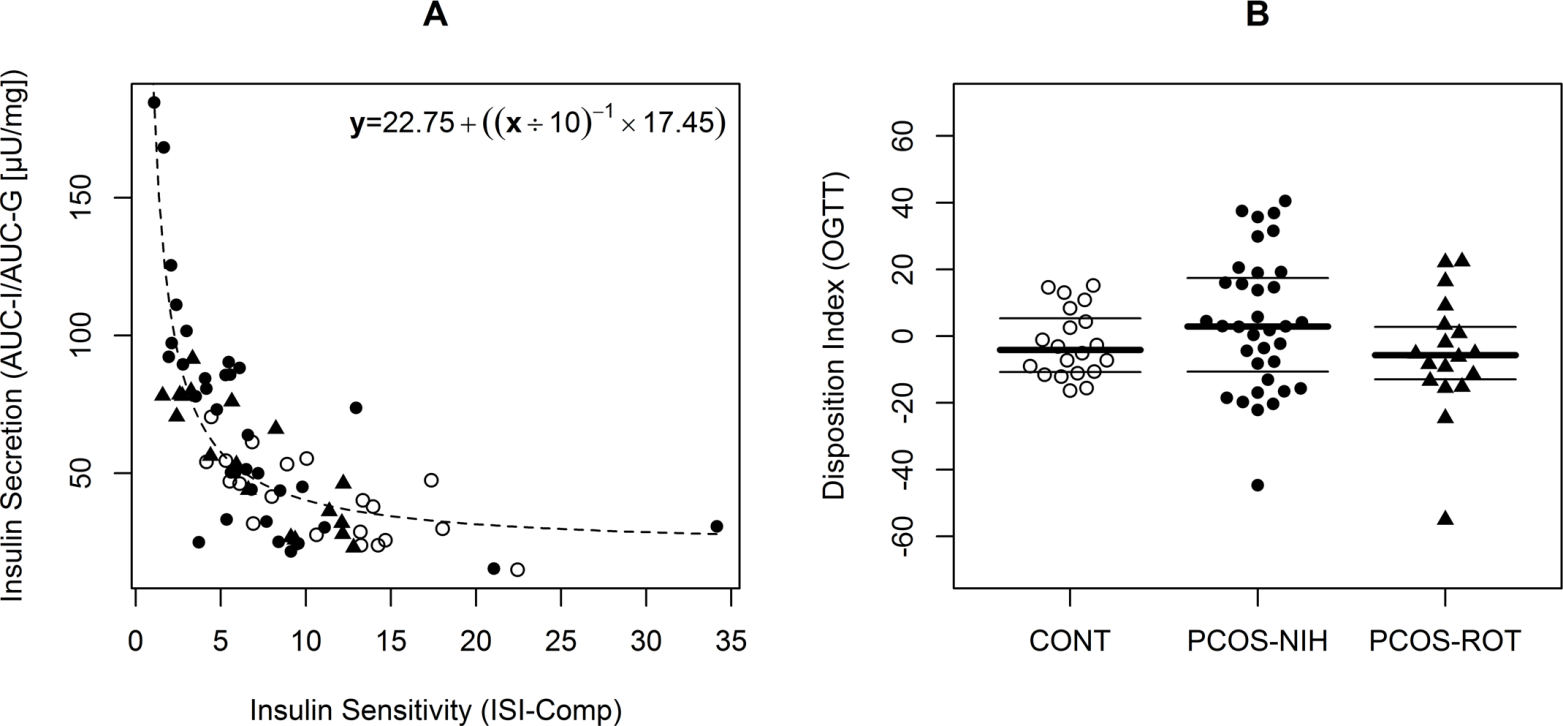
Association of insulin sensitivity with insulin secretion (A). The oral disposition index (i.e. difference between observed and estimated values) in controls (CONT), patients with PCOS classified by NIH 1999 criteria (PCOS-NIH) as well as patients with PCOS additionally classified by the Rotterdam 2003 criteria (PCOS-ROT) (B). CONT, PCOS-NIH and PCOS-ROT are represented as open circles, filled circles and filled triangles, respectively. Lines indicate first, second (median) and third quartiles.

The relation between impaired insulin sensitivity and ß-cell secretion is visualized in Fig 2: The higher degree of insulin resistance in females with PCOS was compensated by an increased pancreatic insulin release. Hence, euglycemia was maintained and the oral disposition index was comparable between the three groups. As provided in Table 3, the compensatory hyperinsulinemia was mainly affecting the later OGTT period, whereby total insulin release was particularly increased in the PCO-NIH subgroup. Moreover, both PCOS subgroups suffered from attenuated hepatic insulin extraction (Table 3).

### Assessment of ectopic lipid content

**Fig 3:**
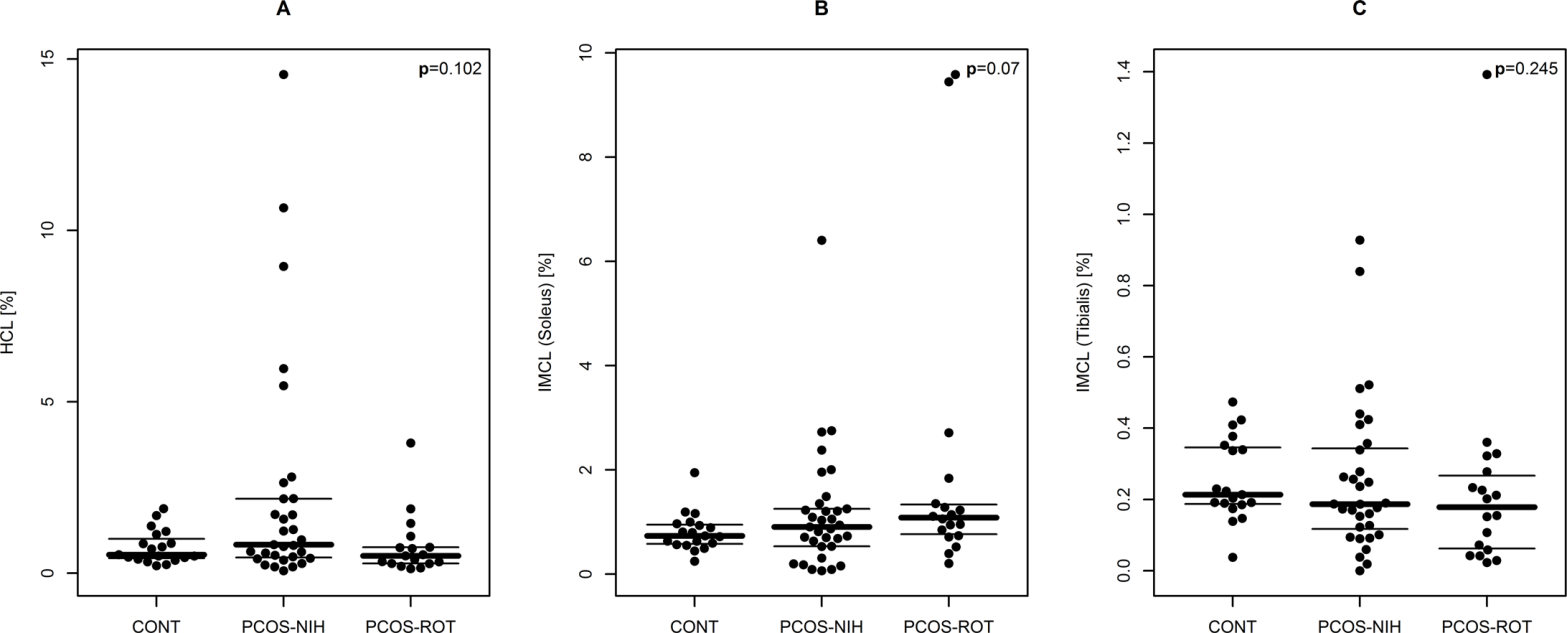
Bee swarm plot of ectopic lipids in different subgroups: controls (CONT), females with PCOS classified by NIH 1999 criteria (PCOS-NIH) as well as females with PCOS additionally classified by the Rotterdam 2003 criteria (PCOS-ROT): A: Hepatocellular lipids (HCL), B: intramyocellular lipids (IMCL) in soleus muscle, C: intramyocellular lipids (IMCL) in tibialis muscle. Lines indicate first, second (median) and third quartiles.

As visualized in Fig 3, no significant differences between PCOS and controls (or between PCOS subgroups) were observed for ectopic lipid content in both liver and skeletal muscle cells. However, correlation analysis revealed that particularly hepatocellular lipids were closely associated with insulin resistance and particularly with OGTT plasma glucose levels in females with PCOS. Hepatocellular lipids were additionally related to dyslipidemia, parameters of body composition, but also to FAI and SHBG. Consistently, the fatty liver index including the combined information from body-composition and hyperlipidemia at fasting state performs well to explain hepatic lipid content (rho = 0.52, p<0.001). The amount of correlation between glucometabolic parameters and cardiovascular risk factors was much lower for intramyocellular lipid content as compared to hepatocellular lipids.

**Fig 4:**
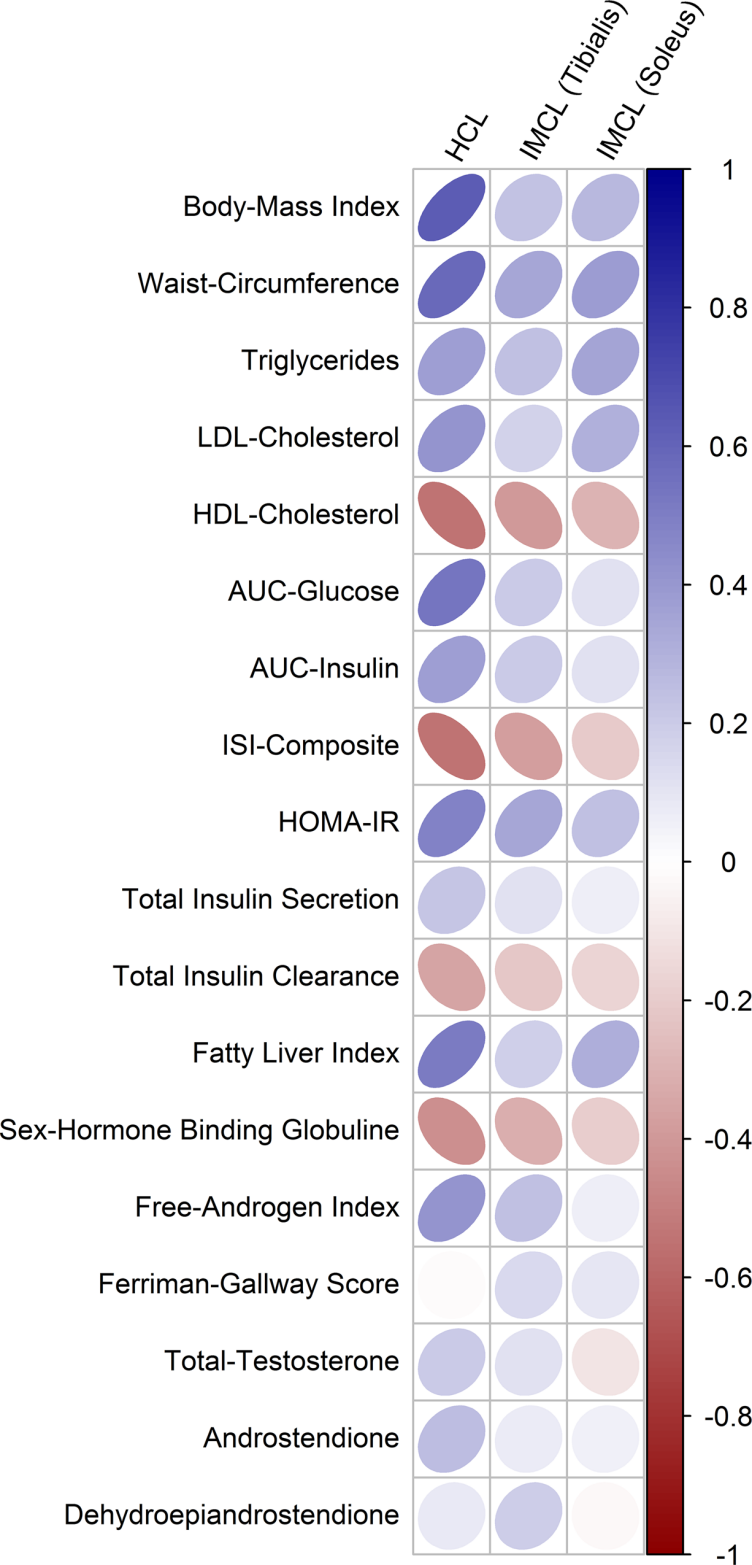
Correlation map representing the amount of association of clinical and metabolic parameters with hepatic (HCL) and intramyocellular (IMCL) lipid content of females with PCOS. The magnitude of correlation is indicated by the shape of the ellipses (circle represents no correlation) and color (dark color represents higher correlation; positive = blue, negative = red).

The associations between clinical and metabolic parameters and ectopic lipids are visualized in Fig 4. Details of the correlation analysis (including correlation coefficients and p-values) are provided in Table 4. A sensitivity analysis of group based comparisons after excluding n = 10 cases using systemic contraceptives is additionally provided (Table 5).

**Table 4:** Correlation analysis representing the amount of association of clinical and metabolic parameters with hepatic (HCL) and intramyocellular (IMCL) lipid content in females with PCOS.

	HCL		IMCL-Tibialis		IMCL-Soleus	
	rho	p-value	rho	p-value	rho	p-value
Body-Mass Index	**0.63**	**<0.001**	0.24	N.S.	**0.28**	**0.047**
Waist-Circumference	**0.59**	**<0.001**	**0.34**	**0.016**	**0.39**	**0.005**
Triglycerides	**0.38**	**0.008**	0.25	N.S.	**0.35**	**0.012**
LDL-Cholesterol	**0.42**	**0.003**	0.17	N.S.	**0.31**	**0.028**
HDL-Cholesterol	**-0.55**	**<0.001**	**-0.40**	**0.004**	**-0.29**	**0.037**
AUC-Glucose	**0.53**	**<0.001**	0.21	N.S.	0.12	N.S.
AUC-Insulin	**0.37**	**0.010**	0.21	N.S.	0.12	N.S.
ISI-Composite	**-0.55**	**<0.001**	**-0.37**	**0.007**	-0.20	N.S.
HOMA-IR	**0.48**	**<0.001**	**0.35**	**0.014**	0.24	N.S.
Total Insulin Secretion	0.23	N.S.	0.12	N.S.	0.07	N.S.
Total Insulin Extraction	**-0.36**	**0.012**	-0.22	N.S.	-0.17	N.S.
Fatty-Liver Index	**0.52**	**<0.001**	0.18	N.S.	**0.31**	**0.034**
Free-Androgen Index	**0.42**	**0.003**	0.24	N.S.	0.07	N.S.
Sex-Hormone Binding Globuline	**-0.43**	**0.002**	**-0.31**	**0.031**	-0.20	N.S.
Ferriman-Gallway Score	-0.01	N.S.	0.15	N.S.	0.09	N.S.
Total-Testosterone	0.20	N.S.	0.12	N.S.	-0.10	N.S.
Androstendione	0.25	N.S.	0.07	N.S.	0.06	N.S.
Dehydroepiandrostendione	0.08	N.S.	0.20	N.S.	-0.03	N.S.

**Table 5:** Sensitivity analysis excluding 10 controls with systemic contraceptive agents during the study period.

	PCOS-Total	PCOS-NIH	PCOS-ROT	Controls	p-value

Age (years)	25 (22–31)	25 (22–29)	26 (22–33)	24 (23–24)	0.273
BMI (kg/m2)	25 (22–30)*	25 (23–30)*	23 (21–30)	21 (21–23)	0.010
HCL (%)	0.73 (0.37–1.71)	0.83 (0.46–2.17)	0.51 (0.28–0.75)	0.70 (0.42–1.07)	0.098
IMCL-Soleus (%)	0.96 (0.66–1.32)	0.91 (0.53–1.25)	1.09 (0.77–1.33)	0.70 (0.56–1.12)	0.287
IMCL-Tibialis (%)	0.19 (0.10–0.31)	0.19 (0.12–0.34)	0.18 (0.06–0.27)	0.28 (0.19–0.35)	0.297
HOMA-IR	1.6 (1.0–2.4)*	1.6 (1.0–2.3)	1.7 (0.9–2.5)	0.7 (0.5–1.6)	0.057
ISI-Comp	5.9 (3.3–9.1)*	5.6 (3.6–8.5)*	6.3 (3.3–10.9)*	12.0 (7.2–14.5)	0.003
Sec-Early (μU/mg)	113 (64–200)	113 (60–215)	113 (70–145)	89 (65–118)	0.250
Sec-Late (μU/mg)	62 (42–88)*	63 (43–88)*	58 (41–86)*	42 (33–49)	0.004
Sec-Total (μU/mg)	56 (32–84)*	64 (33–89)*	55 (33–78)*	39 (29–45)	0.004
FHIE (%)	0.91 (0.90–0.93)*	0.91 (0.90–0.93)*	0.91 (0.90–0.92)*	0.94 (0.92–0.95)	0.021
THIE (%)	0.84 (0.79–0.87)*	0.84 (0.79–0.86)*	0.85 (0.78–0.88)*	0.89 (0.88–0.89)	<0.001

Data are median and interquartile range (IQR) for controls, patients with PCOS (total group) as well as for subgroups classified by NIH 1999 criteria (PCOS-NIH) and patients additionally identified by the Rotterdam 2003 criteria (PCOS-ROT). BMI, body mass index; HCL, hepatocellular lipids; IMCL, intramyocellular lipid content; HOMA-IR, homeostatic model assessment of insulin resistance; ISI-Comp, Composite index; early (sec-early: Δinsulin 0–30 min/Δglucose 0–30 min), late (sec-late: AUC-Insulin/AUC-Glucose [60–120 min]) and total insulin secretion (sec-total: AUC-Insulin/AUC-Glucose [0–120 min]); FHIE, hepatic insulin extraction at fasting; THIE, total hepatic insulin extraction during the OGTT.

* vs. controls: p<0.05.

### Comparison of PCOS phenotypes vs. controls

**Fig 5:**
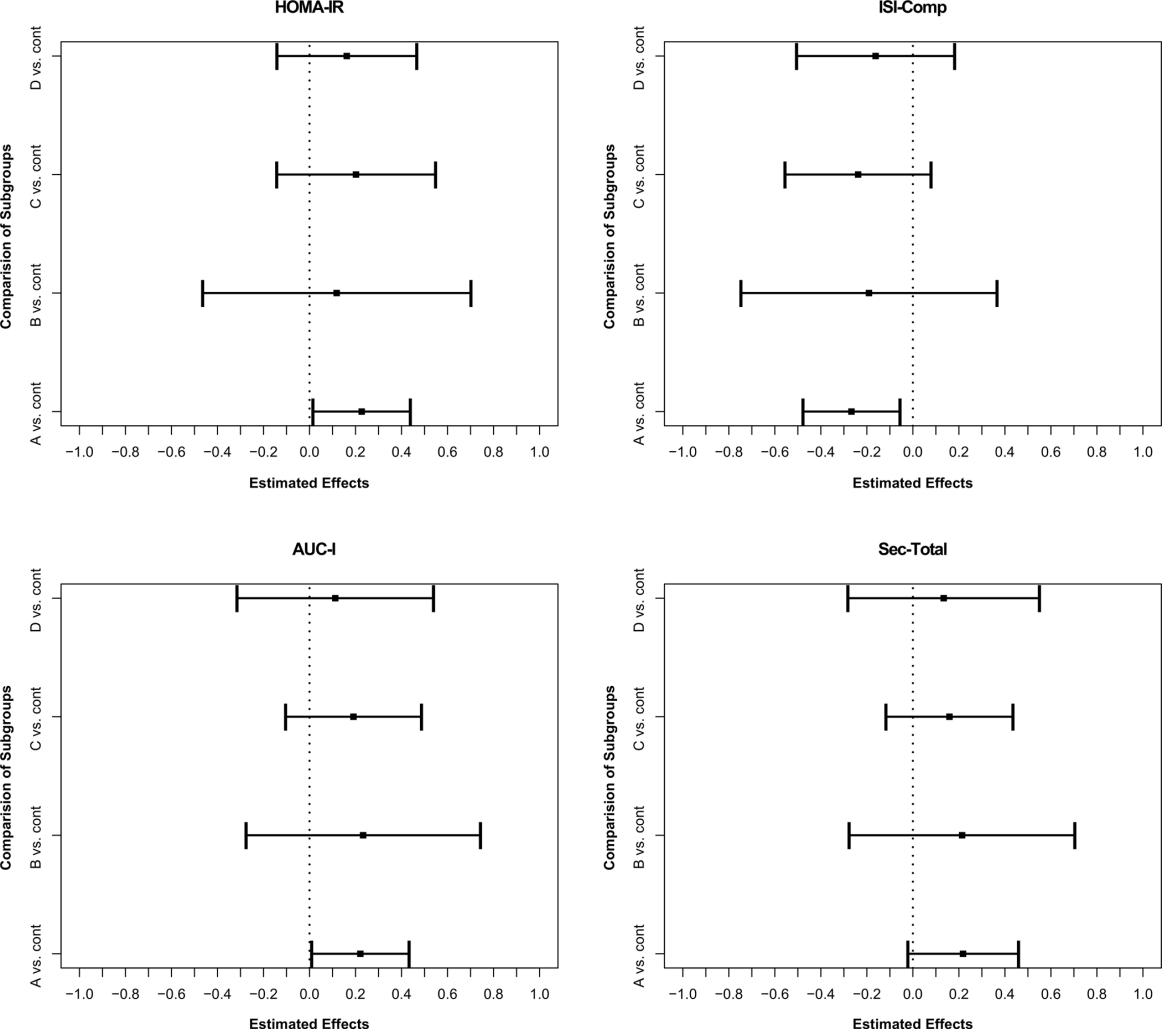
Estimator of nonparametric relative effects and 95% simultaneous confidence intervals (PCOS phenotypes vs. subgroups) for HOMA-IR (homeostatic model assessment of insulin resistance); ISI-Comp (Composite index), AUC-I (area under the concentration curve of insulin) as well as Sec-Total (total insulin secretion) for PCOS subgroups and controls, respectively. A: ovulatory dysfunction + hyperandogenism + polycystic ovaries (n = 28); B: ovulatory dysfunction + hyperandrogenism (n = 7); C: ovulatory dysfunction + polycystic ovary (n = 11); D: hyperandrogenism + polycystic ovary (n = 7); cont: controls (n = 20).

A comparison of insulin sensitivity and insulin secretion between all possible PCOS phenotypes and controls is provided in Fig 5. Phenotypes A (ovulatory dysfunction + hyperandogenism + polycystic ovaries), B (ovulatory dysfunction + hyperandrogenism), C (ovulatory dysfunction + polycystic ovary) and D (hyperandrogenism + polycystic ovary) tend to a higher amount of insulin resistance (i.e. higher scores of HOMA-IR and lower scores of ISI-Comp) and hyperinsulinemia as compared to healthy controls. However, only the comparison of phenotype A vs. controls reached significance (but restricted sample size for some subgroups has to be considered for interpreting these results).

## Discussion

This study aimed to investigate early pathologies in glucose metabolism and their possible associations with ectopic lipids in females with untreated PCOS, categorized by the NIH criteria as well as the additional Rotterdam criteria. While no significant differences between PCOS and controls were observed for ectopic lipid content (i.e. accumulation of lipids in liver and muscle cells), we found that subtle alterations in carbohydrate metabolism are still present in both PCOS classifications.

In particular, females diagnosed by the NIH criteria were characterized by a higher degree of overweight in addition to a more adverse glucometabolic risk profile, with increased dynamic indices of insulin and C-peptide. However, PCOS-NIH as well as PCOS-ROT showed altered shapes of OGTT curves, as they reached the maximum concentrations of glucose and insulin levels significantly later as compared to healthy women, what is comparable to our previous observations in insulin resistant females after pregnancy with gestational diabetes mellitus [29, 30]. Accordingly, both PCOS classifications were affected by impaired insulin action, closely related to obesity as well as androgen excess. While, obesity is strongly contributing to adverse metabolic outcomes in females affected by the disease [31], the association between insulin resistance and PCOS was not fully explainable by the higher degree of overweight or obesity in our study. Likewise, Dunaif et al. found that peripheral insulin sensitivity is decreased about 30–40% in lean and obese females with PCOS (diagnosed by anovulation in addition to hyperandrogenism) using the hyperinsulinemic euglycemic clamp technique [32, 33], what is comparable to the magnitude of insulin resistance observed in subjects with overt type 2 diabetes [6]. Although, our data additionally suggest attenuated insulin action in PCOS-ROT as well, this was not consistently observed in previous studies [6], what might also reflect the lack of clarity to define the clinical features of the disease (i.e. androgen excess but also ovulatory dysfunction and polycystic ovarian morphology) [1, 34].

In any case, impaired insulin sensitivity is a reasonable explanation for the increased incidence of impaired glucose tolerance and onset diabetes observed in women with PCOS [6, 35]. However, before hyperglycemia become overt enhanced pancreatic insulin secretion physiologically adapts to progressing insulin resistance in order to maintain euglycemic conditions as long as possible [36]. To further examine this issue, several studies have assessed ß-cell function in females with PCOS, although with inconsistent results. Dunaif et al. (1996) reported impaired ß-cell function in lean and obese women with PCOS by using the FSIGT (frequently sampled intravenous glucose tolerance test) method, whereby the disposition index was calculated as the product of (minimal model derived) insulin sensitivity and acute insulin response to glucose (AIRg, a parameter reflecting first phase insulin secretion) [37]. More recently, Manco et al. concluded that young women with PCOS but normal glucose tolerance are likely able to compensate for their higher degree of insulin resistance (in terms of ISI-Comp and total insulin secretion during the OGTT) indicating no evidence for ß-cell failure in these subjects [38]. This latter observation is clearly in context with our study as total post load insulin secretion was markedly increased, particularly in females diagnosed according to the NIH criteria. Thus, no differences were observed in the (oral) disposition index. There are two possible explanations for the inconsistent results regarding ß-cell function: First, the number of subjects affected by prediabetic conditions such as impaired glucose tolerance (IGT) was markedly different between the cohorts (3.8% in our study and excluded by [38], whereas 25% of PCOS cases were classified as IGT or diabetic by [37]). As ß-cell function is expected to be impaired in prediabetic conditions the prevalence of IGT has reasonable impact on the results of the studies. Consistently, obese adolescent with PCOS and IGT showed reduced ß-cell function as compared to their normal glucose tolerant counterparts [39]. Second, different methods were used for examining insulin secretion: While AIRg (used by [37]) reflects early insulin response to intravenous glucose load, we found that insulin secretion was particularly increased during the later postprandial period, whereas early secretion was not significantly changed. Moreover, hyperinsulinemia might be additionally triggered by impaired insulin action due to decreased insulin extraction in the liver [6]. Accordingly, our data indicate a lower degree of hepatic insulin extraction in PCOS as compared to insulin sensitive controls.

However, the exact pathophysiological mechanisms beyond altered insulin signaling in females with PCOS is not fully explained. Activation of phosphatidyl inositol 3-kinase was shown to be blunted in PCOS due to serine phosphorylation of insulin receptor substrate-1 (IRS-1) [6]. Regardless of PCOS onset, increased serine phosphorylation of IRS-1 was shown to be mediated by elevated intramyocellular lipids, which are promoted by energy intake, dysfunctional adipocytes as well as impaired mitochondrial function or biogenesis [10, 11, 12]. In view of their possible role beyond the loss of insulin action, special attention of the present study was paid on the assessment of ectopic lipid content in PCOS. In line with the increased prevalence of NAFLD in affected females [13, 14, 15], we hypothesized increased intracellular lipid storage in insulin sensitive tissues might be a plausible explanation for the increased metabolic alterations reported for some PCOS phenotypes. Actually, we were not able to confirm this hypothesis in our study population of untreated women with PCOS, regardless of its definition. In contrast to our results, Jones et al. reported increased liver fat particularly in hyperandrogenic PCOS phenotypes by examining hepatocellular lipids in n = 29 women with PCOS and n = 22 controls by NMR spectroscopy [16]. However, the study participants tended to be older and more obese as compared to our study population. As we found that particularly hepatic lipids were significantly associated with insulin resistance, plasma glucose concentrations, BMI, dyslipidemia, but also decreased SHBG and FAI in PCOS, we suggest that ectopic lipid storage increases along with proceeding metabolic alterations during the later course of the disease. Thereby, the fatty liver index (combined information of body composition and hyperlipidemia) might be a useful tool for an early risk stratification or for follow-up examinations of affected subjects. However, long-term observations are at need to further clarify this topic.

Advantages and limitations of the study have to be discussed: The large number of NMR spectroscopy data is a clear advantage. While subjects with PCOS were untreated, n = 10 controls used systemic hormonal contraceptive agents during the study period. We performed a sensitivity analysis by excluding these subjects to rule out a potential source of bias, however, our basic conclusions (higher degree of insulin resistance in both PCOS subgroups as well as no differences in ectopic lipids) remained unchanged. It might be additionally criticized, that carbohydrate metabolism was examined by OGTT surrogate parameters instead of using the euglycemic clamp or the FSIGT technique. While the OGTT is not yet regarded as the most recommended method to assess the degree of insulin resistance, it represents the gold standard to classify IGT and type 2 diabetes. In addition, dynamic OGTT measurements represent a more physiological examination of postprandial carbohydrate metabolism, closely related to the underlying pathophysiological components of subtle and overt hyperglycemia [40]. Moreover, the sample size of our study cohort is restricted for some subgroup analyses–mainly for the comparisons of PCOS phenotypes vs. controls.

Taken together, we conclude, that the higher degree of insulin resistance (observed for both PCOS classifications) reflects a predominant source of hyperinsulinemia (due to increased secretion or attenuated hepatic insulin extraction), associated with further metabolic and reproductive features of PCOS. In accordance with previous observations [41] we found that females meeting the NIH definition showed a more adverse metabolic risk profile with higher degree of abdominal adiposity and hyperinsulinemia, underlining the possible importance of insulin sensitizers in the clinical management of this subgroup. We were not able to identify significant group specific differences in ectopic lipid content, possibly indicating that increased fat storages in liver and muscle cells rather play a secondary role in the initial pathogenesis of PCOS. However, as particularly liver fat was tightly associated with an adverse metabolic risk profile and hyperandrogenemia in affected females, we suggest that ectopic lipid content might increase (along with proceeding metabolic and hormonal alterations) during the later course of the disease. While there is clear need for long-term observations, consistent algorithms for definition and treatment of PCOS are still missing and need to be developed.
